# Listening Niches across a Century of Popular Music

**DOI:** 10.3389/fpsyg.2017.00431

**Published:** 2017-04-05

**Authors:** Carol Lynne Krumhansl

**Affiliations:** Department of Psychology, Cornell University, IthacaNY, USA

**Keywords:** dehumanization, reminiscence bump, music technology, popular music, music and emotion, age cohort, music decade

## Abstract

This article investigates the contexts, or “listening niches”, in which people hear popular music. The study spanned a century of popular music, divided into 10 decades, with participants born between 1940 and 1999. It asks about whether they know and like the music in each decade, and their emotional reactions. It also asks whether the music is associated with personal memories and, if so, with whom they were listening, or whether they were listening alone. Finally, it asks what styles of music they were listening to, and the music media they were listening with, in different periods of their lives. The results show a regular progression through the life span of listening with different individuals (from parents to children) and with different media (from records to streaming services). A number of effects found in previous studies were replicated, but the study also showed differences across the birth cohorts. Overall, there was a song specific age effect with preferences for music of late adolescence and early adulthood; however, this effect was stronger for the older participants. In general, music of the 1940s, 1960s, and 1980s was preferred, particularly among younger participants. Music of these decades also produced the strongest emotional responses, and the most frequent and specific personal memories. When growing up, the participants tended to listen to the older music on the older media, but rapidly shifted to the new music technologies in their late teens and early 20s. Younger listeners are currently listening less to music alone than older listeners, suggesting an important role of socially sharing music, but they also report feeling sadder when listening to music. Finally, the oldest listeners had the broadest taste, liking music that they had been exposed to during their lifetimes in different listening niches.

## Introduction

The survey reported in this article seeks to characterize the contexts, or “listening niches”, in which people hear popular music throughout their lifetimes. It is an extension of a study that investigated autobiographical memories and life-long preferences for music in young adults (Krumhansl and Zupnick, 2013). That study used top *Billboard* hits from five-and-a-half decades, 1955–2009. For each half decade, a clip was made with a compilation of short, recognizable segments of the top two hits from each year. Participants reported the percentage of songs from each half-decade that they recognized, how much they liked the songs, and how highly they rated the quality of the songs. They also reported their emotional response to the songs from each half decade. Finally, they reported whether they had personal memories associated with the songs and, if so, whether these memories were from listening with parents, alone, or with other people while growing up, or listening alone or with other people recently.

All these measures showed the typical increase for music released over the two decades of their lives, with the highest ratings for the music of the most recent half decade. This is consistent with previous studies showing preferences for music from late adolescence and early adulthood (Holbrook and Schindler, 1989; Schulkind et al., 1999; Janssen et al., 2007). More generally, the term “reminiscence bump” has been used to describe the peak in autobiographical memories and knowledge of events occurring during this period of people’s lives (Rubin et al., 1986). However, we found an unexpected effect in as much as the same measures peaked for the music of their parents’ late adolescence and early adulthood, music of the 1980s. In other words, they were familiar with, and liked, the music that was popular when their parents were the same age as they are now. We knew from their reports that they were listening to the music of the 1980s with their parents, but were not listening to it currently. We called the effect the “cascading reminiscence bump”.

These results suggested it would be interesting to investigate in more detail the contexts in which people of different birth cohorts have listened to and developed preferences for music throughout their lives. The sample includes nearly 1900 participants born between 1940 and 1999, divided into six birth cohorts, those born in the 1940s, 1950s, 1960s, 1970s, 1980s, or 1990s. A short segment was extracted from the most popular song from each year from 1910 to 2009 (based on Whitburn, 1999, for years before 1955, and the *Billboard’s* year-end Hot 100 chart for years since). Ten excerpts were joined together to form a clip for each of 10 decades: 1910s, 1920s, …, 1990s, 2000s.

For the clip of music from each decade, the participants reported whether they knew the songs, whether they liked the songs, what their emotional reactions to the songs were, and whether they had they had personal memories associated with the songs. If so, they were asked how specific the memory is and with whom they were listening. Because the sample of participants varied widely in age, the choices included parents, siblings and other family members, friends and peers, spouses or partners, children, and listening alone. To understand more about the contexts in which they were listening to music, they were asked what styles of music they were listening to during three periods of their lives: growing up, ages 18–25, and now. For the same three periods, they were also asked with what music media they were listening. Because the music spanned a century, the choices included radio, record, tape cassette, dances and parties, concerts, performed by others or by themselves, CDs, and various digital media other than CD, such as digital download and streaming.

Music information systems currently being developed promise new insights into how music is consumed, chosen and distributed, who listens to what styles of music, and how people share information about music with one another. Potentially, this kind of information may provide new information about fundamental issues that have been studied in music psychology. These issues include which aspects of musical structure contribute to memory and preference (e.g., Krumhansl, 1990; Narmour, 1990; Pearce and Wiggins, 2012), how personality traits and context affect musical choices (e.g., Hargreaves and North, 1997; Rentfrow and Gosling, 2003; Gabrielsson, 2011), and the nature of and mechanisms generating musical emotions (e.g., Blood and Zatorre, 2001; Sloboda and O’Neill, 2001; Krumhansl, 2002; Juslin and Västfjäll, 2008; Eerola and Vuoskoski, 2010). Practical insights about the therapeutic use of music and the value of music in public and private spaces may also derive from the analysis of large-scale data on music and its uses.

In particular, streaming services, such as Pandora and Spotify, would seem to greatly expand the amount of data on musical behaviors potentially available. Spotify, in particular, stresses a data-based culture for understanding music behavior, consumption, and choice. These services offer access to huge libraries of music and provide tools to aid listeners’ discovery of new music. Luck (2016) identified psychological factors that make such services attractive, including freedom from ownership responsibility, enhanced discovery and emotional engagement, and nostalgia-fulfilment. However rich the potential of such information, there are limitations. A poll conducted by CivicScience in 2015 showed that 45% of Pandora and 62% of Spotify active users are less than 30 years old^1^. In addition, given the emphasis on discovering new music, the services tend to feature recent, innovative styles. It is hoped that the results of this broad, retrospective survey reported here can be seen as complementing what we can learn from contemporary music information systems.

## Materials and Methods

### Stimulus Materials

**Appendix A** lists the 100 songs that were used to make up the 10 clips that the listeners heard. For the years 1910–1954, before *Billboard* magazine began publishing the year-end Hot 100 chart, the song that was used in the clip was the top single listed in Joel Whitburn’s (1999) *A Century of Pop Music*. His criteria for choosing the top single varied depending on the year. The number of sources and the size of the charts varied, but for each year Whitburn listed the total number of weeks the song appeared on any one of the charts. We chose for each year the song that charted for the greatest number of weeks. For the years 1955–2009, the song was the top single from every year-end Hot 100 chart^2^. These more recent Billboard charts are compiled from national samples of radio air-play, top 40 radio playlists, retail sales and, more recently, internet sales reports.

There were 10 clips, each spanning a 10-year period, with an excerpt from the top song for each year. The excerpts were taken from the songs’ choruses to maximize recognition. Thus, there were a total of 10 songs per clip for each of 10 music decades (1910–1919, 1920–1929, …, 2000–2009). Musical clips averaged 56.6 s (*SD* = 18.89). A practice clip consisted of the second most popular songs from 1955 to 1964. All excerpts were recorded from Spotify’s streaming music service with the exception of a couple from the 1910-1919 era, which were taken from Internet Archive^3^.

### Procedure

The experiment was designed with the *Qualtrics* research suite of tools and participants linked to the questionnaire by way of the Cornell Music Cognition^4^. **Appendix B** lists the questions asked in the survey. After each clip, participants reported the percentage of songs they recognized and how much they liked the songs. All responses were given on a Likert-type scale (0–10), except for the percent recognized (0–100). Participants also rated their emotional responses: sad, happy, nostalgic, romantic, and energized (with 0 = *Does not describe my feelings*, 10 = *Describes my feelings*). Next, they were asked if they would choose to hear similar songs, if given the opportunity. This was included to be a measure of the appeal of the songs from that decade independently of whether or not they were previously familiar with them. Finally, participants reported whether they had personal memories associated with the music. If so, then they were asked how specific are the memories on a scale from 0 to 10, from what period in their life (childhood up to 13 years old, teens ages 13–19, 20s, 30s, 40s, ages 50–65, over 65) and in what social context (listening alone, with parents, spouse/partner, children, siblings or other family members, and friends or peers). For these, they could select all that apply. They first made these responses with the practice clip, and then the 10 clips for each of the 10 decades which were presented in random order.

Following the ratings of the music clips, the participants answered a number of demographic questions: gender, year born, year mother born, year father born, years when children (if any) were born, their nationality, and the country in which they are currently living and, if they were living in the USA, for how many years.

Finally, a number of questions inquired about their music listening histories for each of three periods of their lives: growing up, ages 18–25, and now. For each of these periods, they indicated how many hours they listened to these styles: pop and rock, rhythm and blues, country and folk, classical, jazz, ethnic and world, and other. Then, for the same period they indicated where they heard popular music with these options: radio, record, tape cassette, dances and parties, concerts, heard performed by family and friends, played myself, CDs, subscription services (e.g., Spotify, Rhapsody, etc.), YouTube, Internet radio (e.g., Pandora), digital download (e.g., mp3), and other. They could select all that apply. They answered all of these questions for growing up, before proceeding to ages 18–25, and then they finally answered these questions for now. The protocol was approved by the Cornell University Institutional Review Board. Participants volunteered, granted their informed consent to record their responses, and were not compensated.

### Participants

1910 (729 Males, 1181 Females) participants voluntarily completed the questionnaire. After the publication of Krumhansl and Zupnick (2013), the results were covered in various press media worldwide. The link to Cornell Music Cognition^4^ was included in the NPR coverage^5^, which is most likely the major source of participants, especially the older participants living in the USA. The majority (1085) were living in the USA, but more than 100 participants came from the Netherlands (268), Mexico (183), and Croatia (139), and it was not possible to determine how they found the link to the questionnaire. The questionnaire was discontinued and the data were compiled in October 2013.

The birth years of the participants ranged from 1928–2001. For the statistical analyses, there were enough participants born in each of six decades: 1940–1949 (*N* = 64), 1950–1959 (*N* = 214), 1960–1969 (*N* = 243), 1970–1979 (*N* = 392), 1980–1989 (*N* = 601), and 1990–1999 (*N* = 384). This gives a total number of 1899 participants included in the data analysis. They will be identified in the figures by the midpoint of the decade of their birth, for example 1945 for those born in the decade 1940–1949, and they will be referred to as the 1940s cohort. For the participants currently residing in the USA, their average birth year was 1973. The average birth year of those currently living outside the USA was 1981. When analyzed separately, it was difficult to separate effects of current residency from effects of age differences, so the two groups will not be separated in the statistical analyses that are reported. The average age of their father when they were born was 30.7 years (range 29.0–32.0), with the youngest fathers for the 60s and 70s cohorts. The average age of their mother when they were born was 28.1 years (range 26.5–29.4), with the youngest mothers for the 60s and 70 cohorts.

**FIGURE 1:**
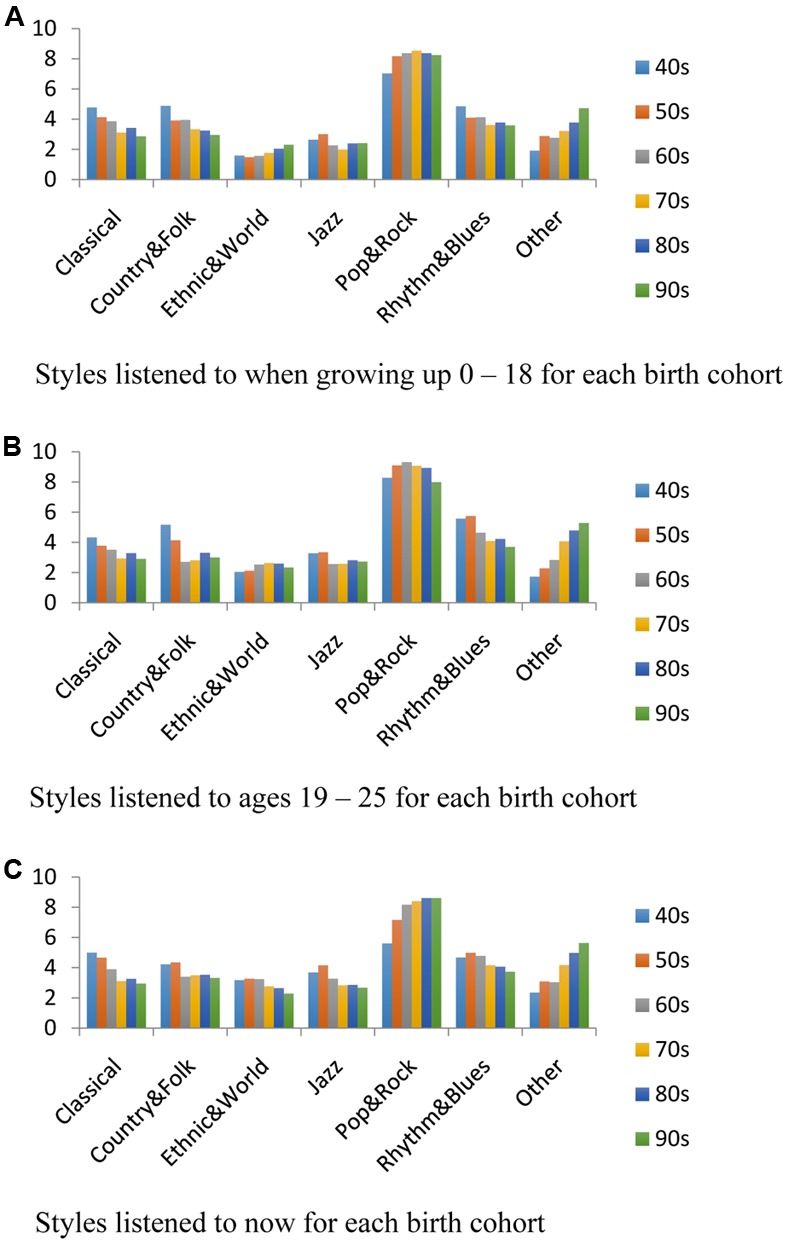
**(A)** Styles of music listened to when growing up for the participants born in each decade, the birth cohorts. **(B)** Styles listened to when ages 19–25. **(C)** Styles listening to now.

**Figure 1** shows the number of hours per week the participants listened to different styles of music. As can be seen, for participants in all cohorts and all three spans of their lives, the most hours were spent listening to rock and pop music. Thus, the focus on *Billboard* top hits in the study was appropriate given their listening histories. The distribution of hours listening across the three time periods of their lives was quite consistent; the correlation between the distributions growing up and ages 18–25 was *r*(5) = 0.97, between growing up and now was *r*(5) = 0.95, and between 18 and 25 and now was *r*(5) = 0.95. Despite these general patterns, some differences were found between the cohorts. The older cohorts listened more to classical, country and folk, and rhythm and blues, whereas the younger cohorts listened more to ethnic and world music, and music that did not fall in any of the categories listed in the questionnaire.

## Results

### Age and Who Was in the Listening Niche

The first analysis was undertaken to get an overview of who was in the participants’ listening niches at different periods of their lives. The data used in the analysis were, for each of six cohorts, how much they were listening to the music of each of 10 music decades (6 cohorts × 10 music decades). This was found for the different periods of their lives (0–12 years, 13–19 years, 20–29 years, 30–39 years, 40–49 years, and 50–65 years); the data for listening when over 65 was too sparse to include. The same data (6 cohorts × 10 music decades) were compiled for whom they were listening to the music with (parents, siblings and other family members, with friends or peers, with spouse or partner, with children, or alone).

**FIGURE 2:**
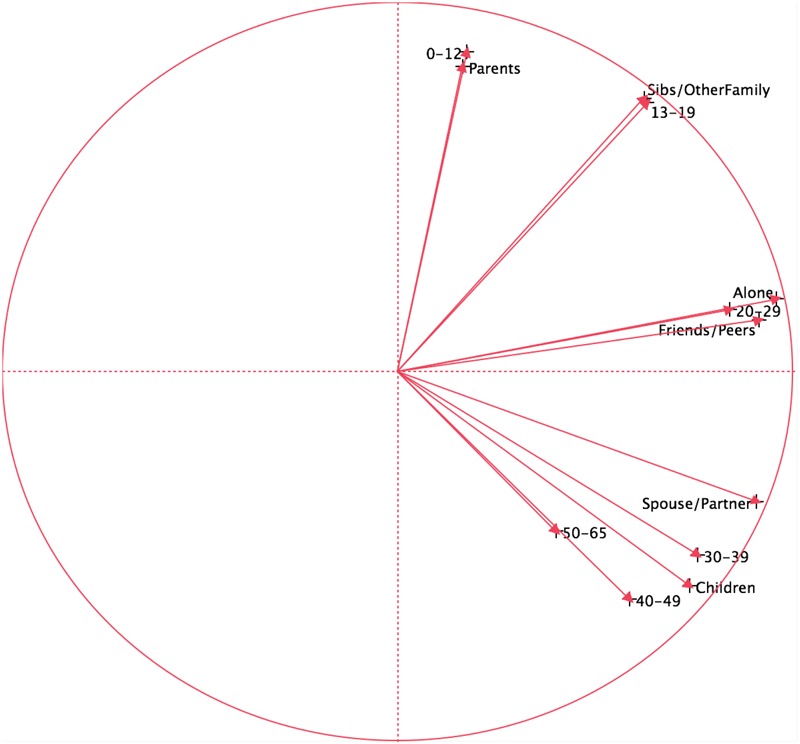
**Principal components analysis of music listened to at different ages and with whom**.

**Figure 2** shows the results of a principal components analysis done on these data. The arrows point in similar directions if they were listening to similar music at these times of their lives with these individuals. It shows that when the participants were ages 0–12, they were most often listening to music with their parents, by ages 13–19, they were listening more with siblings and other family members. Then later, through their 20s, they were more often listening alone or with friends and peers. By ages 30–39, music was listened to with spouse or partner, and then with children for participants in their 30s and 40s. The first (horizontal) dimension accounted for 48.1% of the variance in the data; the second (vertical) dimension accounted for 32.3% of the variance, for a total of 80.4% of the variance. Overall, the results suggest a regular progression of listening with different groups of people throughout the life span ranging from parents in early life to children in later life.

### Song Specific Age

The next analysis looked at the liking ratings as a function of the participants’ age at the time the music was popular, the “song specific age” (Holbrook and Schindler, 1989). It was calculated as the approximate age they were when the song was popular. For example, the song specific age for the cohort born in the 1960s and the music of the 1980s was 20. The analysis was also done on 5-year cohorts, with similar results and will not be reported.

The results showed an increase in how much they liked the music up to the age of about 20 and then a decrease for music that was popular later in their lives. This was confirmed by a polynomial regression which accounted for 62% of the variance [*F*(2,57) = 46.9, *p* < 0.0002] and both the linear and quadratic effects were significant [*F*(1,57) = 45.3 and 48.4, respectively, both *p* < 0.0001). Overall, liking ratings were lowest for the songs that were popular long before the participants were born, and for the most recent songs for those in the oldest age cohort.

**FIGURE 3:**
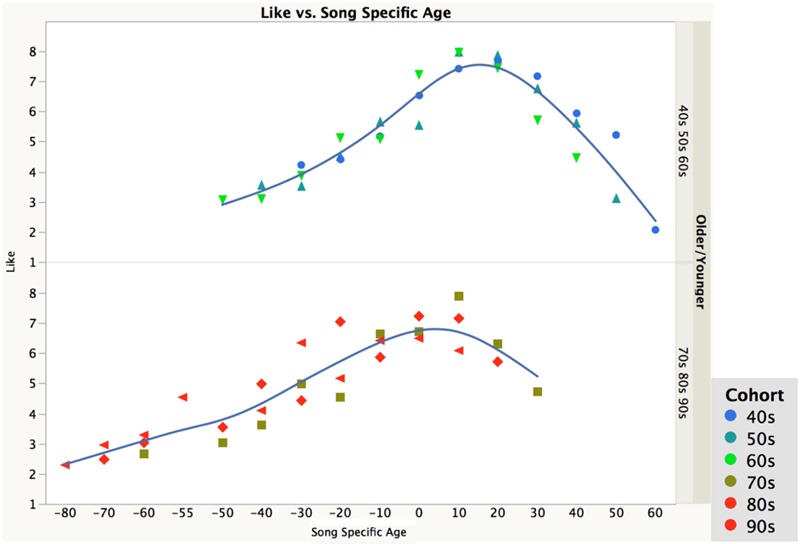
**Plot of liking ratings as a function of song specific age (the age of the participant when the song was popular), divided up between the three oldest and three youngest birth cohorts**.

However, a closer look showed notable differences between the three oldest cohorts (40s, 50s, 60s) and the three youngest cohorts (70s, 80s, 90s). The liking ratings for the two groups as a function of the song specific age are shown in **Figure 3**. It is apparent that the song specific age effect is stronger and more regular for the older cohorts than for the younger cohorts; the peak is more distinct and occurs somewhat later for the older cohorts than the younger cohorts.

### Music Decade

The next analysis considered whether there were overall preferences for different decades of music. To look at this, the decade of music was added to the analysis of variance with linear and quadratic effects of song specific age (as above). In other words, the analysis looked to see whether once the effect of song specific age was factored out there was a residual effect of the decade of the music. The analysis with both the song specific age and decade accounted for 86% of the variance in the liking ratings [*F*(11,48) = 26.6, *p* < 0.0001] and the effect of decade was highly significant [*F*(9,48) = 9.0, *p* < 0.0001].

There were peaks for music popular in the 1940s and in the 1960s. A contrast comparing music from the 1940s to the music from the 1930s and 1950s produced a marginally significant effect [*F*(1,48) = 3.5, *p* = 0.066, which would be significant by a one-tailed test]. A contrast comparing music from the 1960s to the music from the 1950s and 1970s produced a significant effect [*F*(1,48) = 10.4, *p* = 0.0023]. Thus, the peaks for music of the 1940s and 1960s were confirmed statistically. A contrast was also computed testing whether the average liking ratings for music of the 1980s exceeded that for the 1970s or 1990s because the earlier paper (Krumhansl and Zupnick, 2013) found a peak for the music of the 1980s in college age participants. The result was non-significant [*F*(1,48) = 1.74, *p* = 0.19].

**FIGURE 4:**
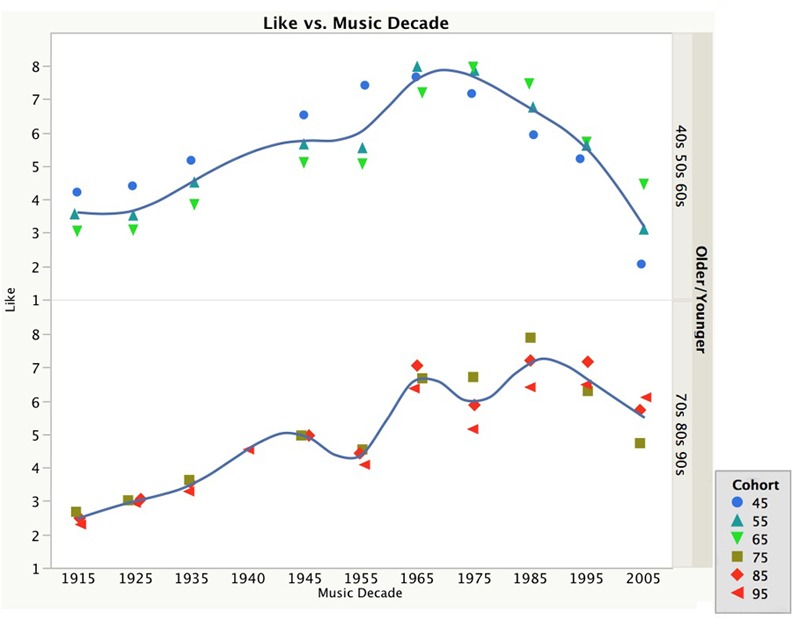
**The liking ratings as a function of the decade of the music, divided up between the three oldest and three youngest birth cohorts**.

However, as can be seen in **Figure 4** the decade of music effect was stronger for the younger cohort than the older cohort. Their liking ratings showed clear peaks for the music in the decades of the 1940s, 1960s, and 1980s. In contrast, the liking ratings for the older cohort were more evenly distributed with a broad peak around the music of the 1960s and 1970s, which is consistent with the song specific age effect described earlier.

### Emotional Reactions

**FIGURE 5:**
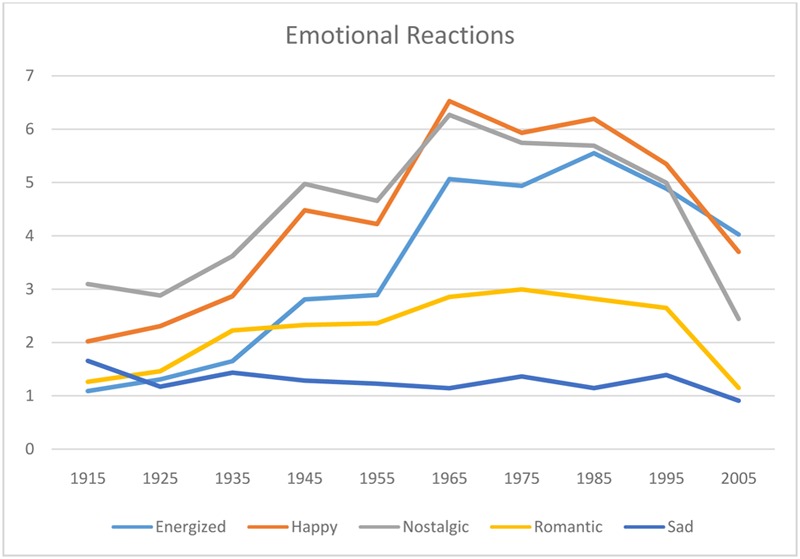
**The emotional reactions to the music of the different decades**.

**Figure 5** shows the emotional reactions to music of the different decades. There was a significant effect of decade for all the emotion scales, with the weakest effect for sad [energized *F*(9,50) = 21.1, *p* < 0.001, happy *F*(9,50) = 18.3, *p* < 0.001, nostalgic *F*(9,50) = 7.7, *p* < 0.001, romantic *F*(9,50) = 12.0, *p* < 0.001, sad *F*(9,50) = 2.8, *p* = 0.01] For all the scales (except sad) there was an increasing trend from the earliest decade to the music of the 1980s, and then a decrease. For sad, a test comparing means showed that the only significant difference is between the 1910s (the saddest) and the 2000s (the least sad). Distinctive peaks relative to neighboring decades can be seen in the curves for happy, nostalgic, and energized for music of the 1940s, 1960s and 1980s (except for nostalgia, possibly because the music is relatively recent).

The next analysis considered how much the emotional reactions accounted for how well they liked the music. A multiple regression predicting liking from these five emotional responses accounted for 99.2% of the variance [*F*(5,54) = 1346.9, *p* < 0.0001], which indicates that the emotional reaction to the music is a very strong predictor of how well the music is liked. Each of the five emotions was significant in the multiple regression [energized *F*(1,54) = 5.20, *p* = 0.03; happy *F*(1,54) = 42.7, *p* < 0.0001, nostalgic *F*(1,54) = 40.2, *p* < 0.0001, romantic *F*(1,54) = 16.7, *p* < 0.0001, sad *F*(1,54) = 38.0, *p* < 0.0001], suggesting they are each making independent contributions to how well the music is liked. The regression coefficient for all of the emotions except sad was positive, suggesting that sadder popular music is less preferred. It should be noted, however, that the music from none of the decades is rated highly on sad.

Because the liking ratings might be influenced by whether the participants recognized the songs (and the correlation between the two was, in fact, *r*(58) = 0.92, *p* < 0.0001), the survey included another question about whether they would choose to hear music like that in each decade again. The correlation with whether they recognized the music and whether they would like to hear music like that again was still fairly strong [*r*(58) = 0.87, *p* < 0.0001]. However, there was a possibly interesting difference in the emotions that predicted whether they said they would like to hear music like that again. The five emotion ratings accounted for 98.2% of the variance [*F*(5,54) = 607.4, *p* < 0.0001], but only happy and romantic contributed positively [happy *F*(1,54) = 97.4, *p* < 0.0001, romantic *F*(1,54) = 6.9, *p* = 0.011] and energized contributed *negatively* [*F*(1,54) = 12.9, *p* = 0.0007]; the other two scales were marginally significant and in the same direction as before. Thus, hearing music that makes the participants feel energized made them less likely to want to hear music like that again.

The final analysis considered whether the different birth cohorts had different emotional reactions to the songs of different decades. Even though the younger participants didn’t know the older songs and the older participants didn’t know the most recent songs, they agreed on their emotional reactions to the music. To look at this statistically, for each birth cohort, an emotion profile was made of the five emotion scales for the 10 decades of music. For example, the emotion profile for the 40s cohort was the rating on the five emotion scales for all 10 decades of music, for a total of 50 values. The correlations between the emotion profiles for all pairs of cohorts were highly significant (at *p* < 0.001, when Bonferonni corrected for multiple comparisons). This might be an artifact of the low ratings on sad, so the same analysis was done after that scale was excluded and the correlations between all pairs of cohorts were still highly significant (except for the correlation between the oldest and the youngest cohorts when corrected for multiple comparisons).

### Personal Memories

Overall, 53.6% of the participants reported having personal memories associated with the songs in the 10 decades, and those memories were rated an average of 5.73 on specificity (0–10). There was no effect of birth cohort on either the percent of associated memories or their specificity. Both measures correlated strongly with whether they liked and recognized the songs, and wanted to hear songs like that again. Listeners’ reported memories correlated most strongly with music they heard when they were 13–19 years old [*r*(58) = 0.81, *p* < 0.0001) and 20–29 years old (*r*(58) = 0.83, *p* < 0.0001], although how much they listened to music from all periods of their lives (except ages 50–65) correlated significantly with the proportion of people reporting associated memories; the same was true for the specificity of the memory.

The incidence of personal memories was also associated positively with the song decades that were rated high on making them feel energized, happy, nostalgic, and romantic [*r*(58) = 0.94, *r*(58) = 0.95, *r*(58) = 0.86, *r*(58) = 0.66, respectively, all *p* < 0.0001], and negatively on sad [*r*(58) = –0.29, *p* = 0.023]. The proportion of participants reporting personal memories correlated most strongly with music they heard listening alone [*r*(58) = 0.96, *p* < 0.0001] and with friends and peers [*r*(58) = 0.91, *p* < 0.0001], the music they heard most often in their teens and early adulthood, but the correlations were significant for all periods of their lives. As for how specific the memories were, the ratings correlated most strongly with music they heard listening alone [*r*(58) = 0.90, *p* < 0.0001] and with friends and peers [*r*(58) = 0.91, *p* < 0.0001], but the correlations were significant for all of the music they listened to with others except for music they listened to with parents.

### Music Media

**FIGURE 6:**
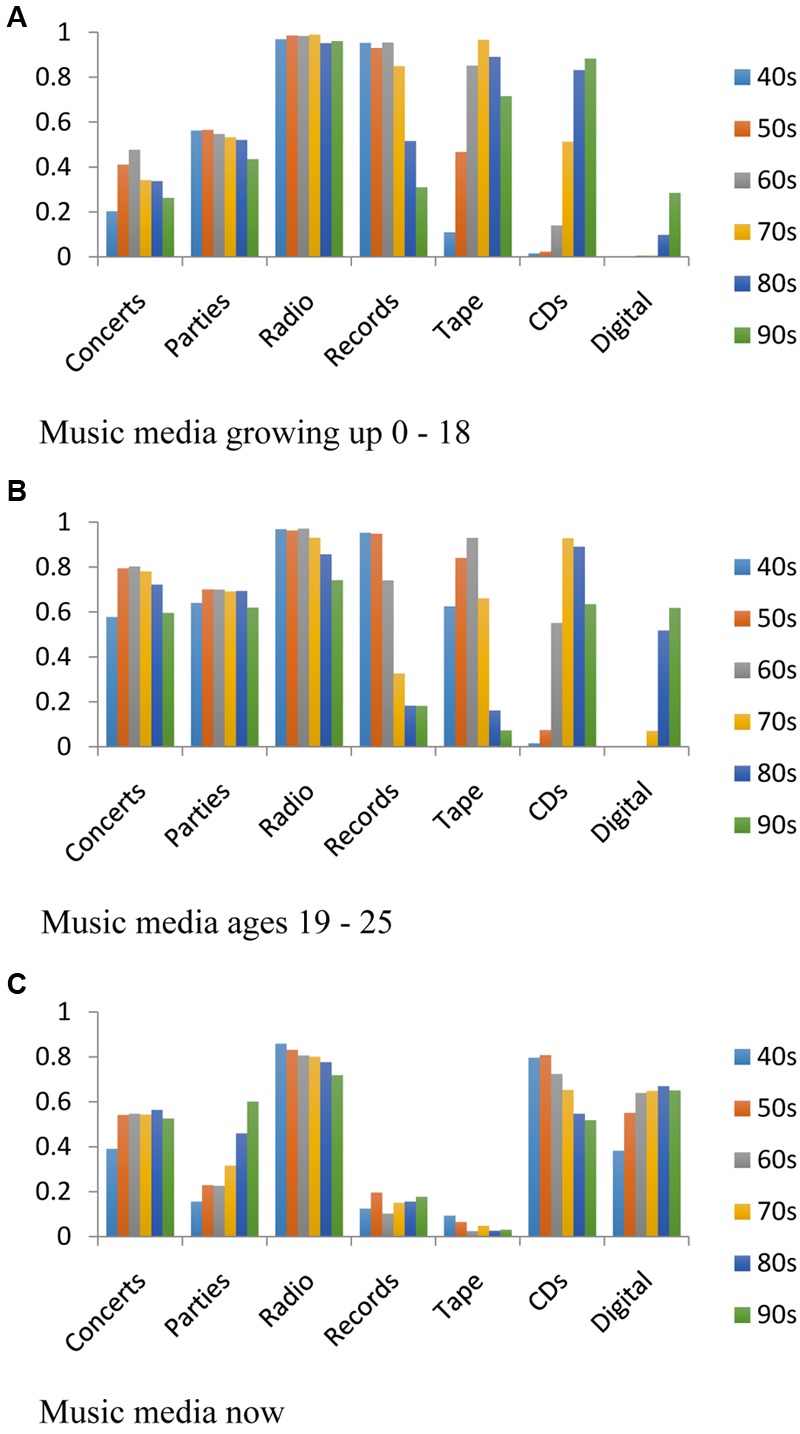
**(A)** The music media used when growing up. **(B)** Media used ages 19–25. **(C)** Media used now.

Participants also indicated which media they were using when listening to music during three periods of their lives: growing up, 19–25 years, and now. **Figure 6** shows the percentage of people in each birth cohort who were listening to music on the most common media: concerts, parties, radio, records, tape, CDs, and Digital. Digital was the composite of digital download, YouTube, internet radio, and subscription services. The responses for “played myself” were not included because of the ambiguity of the question: whether they were performing it themselves, or playing a recording of someone else performing the music.

For all periods of their lives, they were listening to music on radio at a fairly high level although note the decreasing use of radio presently. The youngest birth cohort is listening to music almost as much in digital formats. Beyond that, we see effects of the period of their lives that relate to music media. Growing up, the older participants were listening to music on records, whereas younger birth cohorts were listening to music on tape, and the youngest on CDs and in other digital formats. For music in late teenage and early adult years, the oldest listeners were hearing music on records, but also tapes; the middle birth cohorts had clearly switched to tape, and the youngest participants were listening to music on CDs and on digital media. Finally, nearly no one is listening to music on records or tapes now, but more on CDs and other digital formats, even including the oldest birth cohorts. Finally, participants seem to have heard music at concerts and parties most often when they were ages 19–25 years.

### Differences between Birth Cohorts

The results described above showed that the decade effect (preferences for music of the 1940s, 1960s, and 1980s) was stronger for the younger generations and the song specific age effect (with a peak in preference for music popular in late teens and early 20s) was stronger for the older generations. When looking for other differences between the birth cohorts, some obvious effects emerged. For example, the younger cohorts were less familiar with the older music and liked it less than the more recent music; the opposite was true for the older cohorts. Three less obvious findings emerged, however.

**FIGURE 7:**
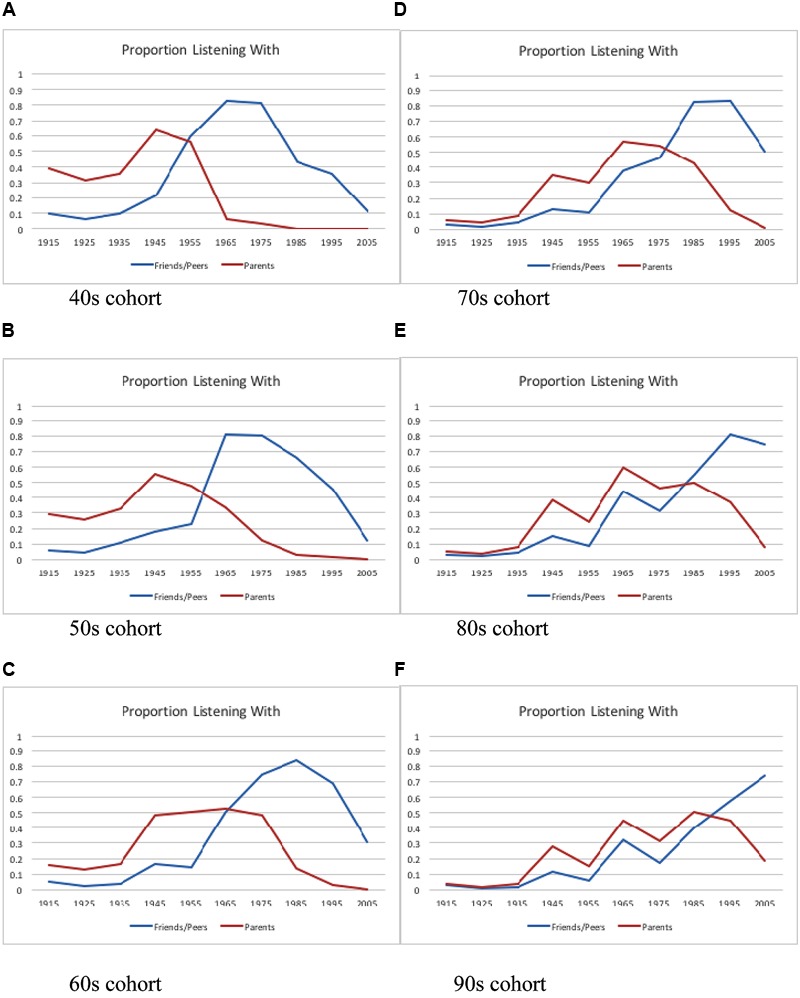
**The decade of the music listened to with friends and parents for participants born in each decade. (A)** 40s cohort; **(B)** 50s cohort; **(C)** 60s cohort; **(D)** 70s cohort; **(E)** 80s cohort; **(F)** 90s cohort.

One finding concerned the overlap between the music they listened to with their parents and their friends and peers. **Figure 7** shows for each cohort the decades of the music they listened to with their parents and their friends and peers. The oldest three birth cohorts listened to the older music with their parents and the newer music with their friends, with very little overlap. When it comes to the cohort born in the 1970s, we start to see them listening to the older music with their parents, particularly the music of the 1940s and 1960s, and only the newer music, the music of their early adulthood, with their friends and peers. This pattern became stronger for the birth cohorts from the 1980s and 1990s.

**FIGURE 8:**
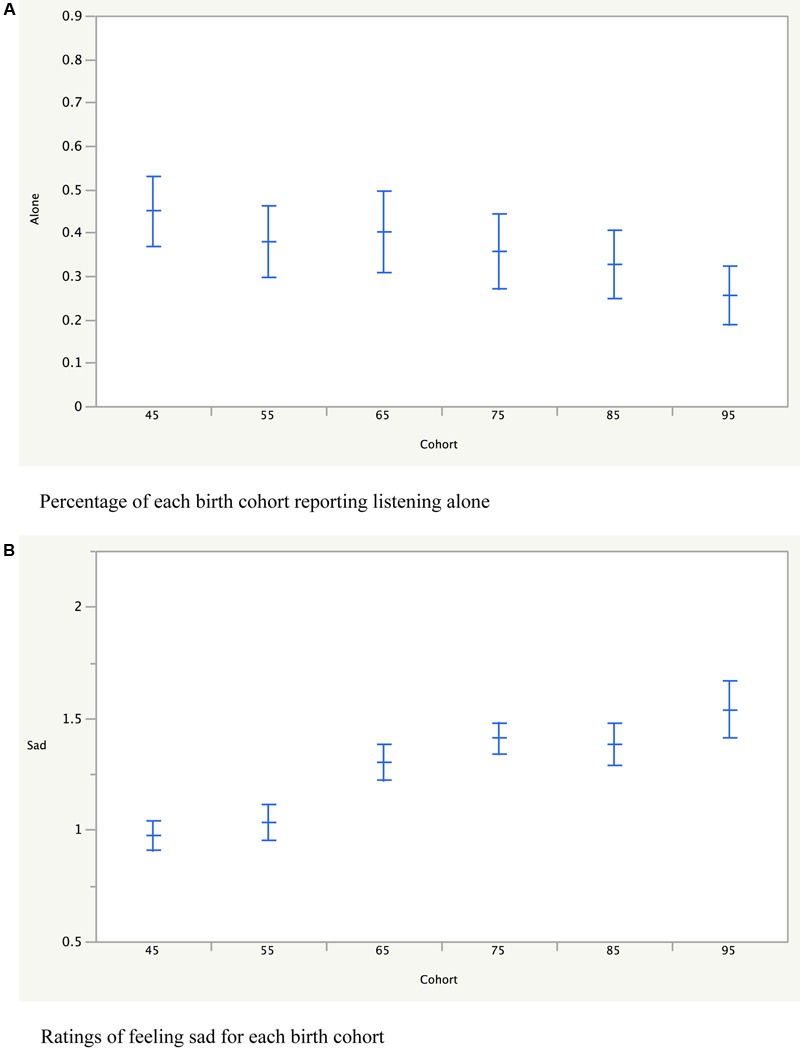
**(A)** Shows for each birth cohort how much music they listened to music alone (average and mean error bars). **(B)** Ratings of each cohort of feeling sad while listening to music (average and mean error bars).

As described earlier, there was a predictable pattern of who was listening to music with the participants as they moved through different stages of their lives, from parents to children. However, there was a somewhat surprising effect of birth cohort on how much they listened to music alone. **Figure 8A** shows the percentage of people in the different birth cohorts who reported listening to music alone, showing a decline for younger participants. When decade of music was included in the analysis, a linear contrast found a quite significant decreasing effect of birth cohort [*F*(1,54) = 10.9, *p* = 0.0017] on how much they were listening to music alone. Given the prevalence in more recent years of personal listening devices, one might have expected the opposite effect.

The final effect concerned the different cohorts’ overall emotional responses to the music. No significant effect of birth cohert was found on any of the emotion scales, with the exception that the younger birth cohorts generally gave notably higher ratings on sad. As can be seen in **Figure 8B**, the younger birth cohorts judged the music of all decades to make them feel sadder than the older birth cohorts [*F*(1,58) = 29.8, *p* < 0.0001].

### Cumulative Effects of Listening Niches on Musical Preferences

**FIGURE 9:**
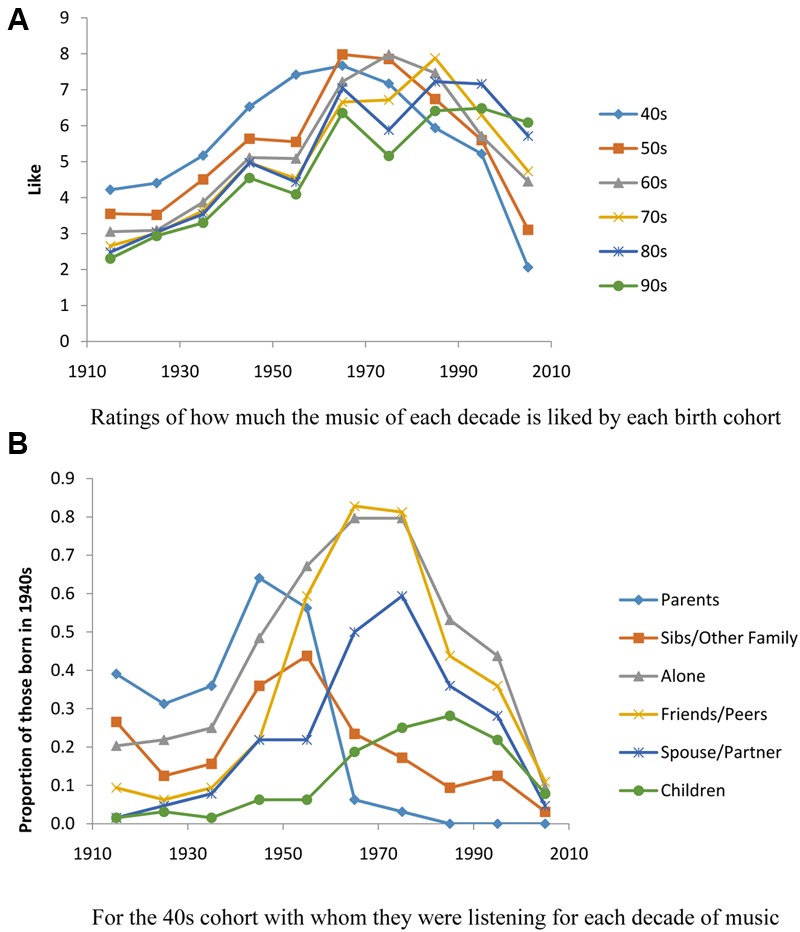
**(A)** The liking ratings of the birth cohorts for each decade of music. **(B)** For the generation born in the 1940s, with whom they were listening for each decade of music.

**Figure 9A** graphs how much each birth cohort liked the music of each decade. As can be seen, those born in the 1940s had a broader liking curve than any of the other birth cohorts. This may be because they have, over their lifetimes, listened to music with more different types of people. **Figure 9B** shows, for each decade of music with whom they were listening. With their parents, they were listening to the music of the 1940s, during the decade in which they were born. They were also listening to music of the 1910s, music that their parents would have been listening to with *their* parents in the decade in which they were born, that is, with our participants’ grandparents. With siblings and other family members, they were listening to this same music and also to the music of the 1950s, music that was contemporary when they were young. The listened alone most to music of the 1950s, 1960s, and 1970s, the music of their teen years, 20s, and 30s. With friends and peers, they listened most to music of the 1960s and 1970s, in their teen and early adult years. Overall this birth cohort listened to music most often during this period of their lives. They listened with spouse or partner most to music of the 1960s and 1970s, when in their 20s and 30s. And, finally, they listened with their children most to music of the 1980s, when their children would have been in their teens. Note that after this they were listening relatively little to newer music, especially music of the 1990s and 2000s. Note that in **Figure 9A**, they liked the music of these decades least, the music that they also were not hearing in any of their listening niches.

## Discussion

The main objective of the present study was to gain a more detailed understanding of the contexts in which people listen to and develop preferences for music. One important component of the “listening niches” was with whom they were listening. Given the wide range of ages of the participants, it was possible to trace a regular progression throughout the life span: they were listening with parents as children, with siblings and other family members in their teen years, with friends and peers and alone in their twenties, with spouse or partner in their thirties, and finally with children when they were in their forties.

The present study replicated the song specific age effect found in many studies (e.g., Holbrook and Schindler, 1989; Schulkind et al., 1999; Schubert, 2016). The effect is an overall preference for songs that were popular in late adolescence and early adulthood. A recent study by Rathbone et al. (2016) found, however, that the reminiscence bump was pronounced only if the music was personally significant to the listener. Other factors that might contribute to the reminiscence bump found for music (and also for other domains, such as public events, sports, and films) include the occurrence of personally significant events during these years, physiological changes, formation of personal values, and music as a badge of social identity (see Rubin et al., 1998, for a review).

Another effect found in this study was a decade effect. Music of the 1940s was preferred to music of its neighboring decades (i.e., the 1930s and 1950s), and the same was true for music of the 1960s. The music of the 1980s also showed a peak, but it was different from its neighbors only for the younger participants. To try to understand the decade effect, the emotional responses to music of the different decades were considered. Consistent with the decade effect, the music of the 1940s and 1960s was judged to make the participants feel happier and more energized and nostalgic than the music of their neighboring decades. The same was true of the music of the 1980s, although the effect of nostalgia was somewhat muted possibly owing to its relative recency. These results are in line with the finding that popular music is generally judged to be positive in both valence and arousal (e.g., Platz et al., 2015).

In general, the popular music used here was not judged to be sad, except perhaps for the oldest decade of music, the 1910s. Schellenberg and von Scheve’s (2012) analysis of 1000 Top 40 recordings found an increase over the period from 1965 to 2009 of minor mode and slower tempo. Consistent with this, the minor mode songs in the present study were predominantly from the most recent decade. However, it might be noted, the study by Platz et al. (2015) did not support the shift to sadder songs over this period in German popular songs; their study included music from the period 1930 to 2010. Schellenberg and von Scheve (2012) hypothesized that this shift to minor mode and slower tempo would make more recent songs sound sadder, although they did not test this empirically. Our participants did not rate the more recent music as making them feel sadder than earlier songs (in fact, none of these top hits were rated as making them feel sad), but they did rate the more recent songs as making them feel less energized, happy, nostalgic, and romantic.

The same influences of emotion were found for personal memories associated with the songs: their incidence was positively related to songs that made them feel energized, happy, nostalgic, and romantic, and negatively to those that made them feel sad. Despite the century long span of the music, more than half the participants reported personal memories associated with the music in the study. This complements the finding that 30% of the time listeners in Janata et al. (2007) study had somewhat or strongly autobiographical memories associated with 1500 randomly selected popular songs. The prevalence and specificity of personal memories were greatest for music heard in the teens and 20s, but also came from all periods of their lives. They were most prevalent and specific for music heard with friends and peers, and alone, but were associated with all contexts, except for music listened to with parents possibly because autobiographical memory emerges gradually in development (Nelson and Fivush, 2004). The older participants judged their personal memories to be as specific as the younger participants, but it should be noted that there are general shifts from episodic to semantic details in autobiographical memories with aging (Levine et al., 2002). Overall, these results are consistent with the frequency, durability, strength and rich content of autobiographical memories associated with music (e.g., Gabrielsson, 2001, 2011; Janata et al., 2007; Belfi et al., 2015).

The emotion rating scales almost perfectly predicted how well the music from the different decades was liked. However, other factors might be involved. In the 1940s, WW II made popular both songs that brought the war home and sentimental ballads for those remaining at home (Sanjek, 1988), which have been absorbed into film and other popular media (Basinger, 2003). After the war, high-quality, low cost tape recorders helped establish independent labels broadening the musical styles available on recordings (Burgess, 2014). The 1960s was a time of political unrest and tremendous artistic innovation, including that of the Beatles and the Rolling Stones, but also Motown, country, folk and, late in the decade, disco and hip hop. The 1980s ushered in a conservative political era and saw the introduction of music videos on MTV, and influential albums by Michael Jackson, Madonna, Springsteen, Prince, and others. Burgess (2014) also details technical advancements in music production during these decades. It is impossible to assess from the current survey how influential these, and cultural and artistic factors, have been in establishing the participants’ preferences and emotional responses.

The survey does, however, provide some information about the media the participants were using to hear popular music. They reported how they were hearing music during three periods of their lives: when they were growing up, when they were 19–25 years of age, and now. Radio has been a major source of music for all birth cohorts during all periods of their lives, although a decline was apparent for the youngest birth cohorts. In the 1940s, the transistor radio was invented, and car radios came in by the late 1940s. In the 1960s, radio developed the long-playing FM format, and AM radio innovated the Billboard Hot 100 in 1959. Internet radio was pioneered in the 1990s. Thus, radio in its various forms has been a constant source of music delivery for all the birth cohorts. Other music media have undergone shifts, however, and this might be a partial cue to the decade effect found.

Important changes in how music could be heard occurred in the 1940s, 1960s, and 1980s (Burgess, 2014). Columbia Records introduced the 33 1/3 RPM long playing record in 1948 with greatly improved signal to noise ratio and longer playing times. The survey found that records were the predominant music media (together with radio) while growing up for the cohorts born in the 1940s, 1950s, 1960s, and even the 1970s, suggesting that young participants were listening to their parents’ music on their parents’ media, records. However, by the time they were listening to music during ages 19–25 they shifted to the new media of the 1960s, tape. Phillips compact cassette was introduced in 1963, making it possible to listen to music almost anywhere and inexpensively sharing it with others. For the participants born in the 60s and 70s, tape was the predominant music media while growing up, again suggesting that they were listening to their parents’ music on their parents’ media, tape. But by the time they were listening to music during ages 19–25 they shifted to the new media of the 1980s, CDs. Sony and Philips introduced the CD format in 1983. For the cohort born in the 1960s, 1970s, and 1980s, tape was still the primary music media while growing up, again suggesting they were listening to their parents’ music on their parents’ media, tape. However, by the time they were 19–25 years of age, they were primarily listening with the new technology, music on CDs.

Stepping away from these particular results, one factor contributing to the preferences for music of the 1940s, 1960s, and 1980s may be the introduction of music media that were significant improvements over previous media. The most likely candidates, based on the survey results, are: long-playing records, cassette tapes, and CDs. While growing up, listeners appear to have heard the music of the previous birth cohorts on the older technologies, but actively sought new music on the new technologies in their teens and twenties. Perhaps it is during that period of their lives that they began building their own music collections in the new media, developing their musical preferences, and establishing associated personal memories and emotional responses. Radio has been a major source of music for all birth cohorts, although the digital formats (other than CD) seem to be overtaking radio for the youngest cohorts. An interesting question, given the adoption of streaming services with no physical musical artifacts (Luck, 2016), is whether intergenerational transfer of music will be less prevalent in the future, or whether the easy access to very large music libraries will actually facilitate sharing music across generations.

Finally, the study turned up some generational differences. Listeners born in the 1940s, 1950s, and 1960s listened to very different music with their parents and their friends. They listened to the older music with their parents, but more contemporary music with their friends. This is consistent with the idea that the older birth cohorts used music, particularly the music of the 1960s and 1970s, to distance themselves from their parents. In contrast, those born in the 1970s, 1980s, and 1990s listened to some of the older music with both their parents and their friends, especially music of the 1940s and 1960s and, for the youngest two birth cohorts, the music of the 1980s, replicating Krumhansl and Zupnick (2013).

Other generational differences were found. The oldest three birth cohorts showed a stronger effect of song-specific age, whereas the youngest three birth cohorts showed a stronger effect of the decade of the music. One possible explanation for this is that the older participants may generally have had less access to a wide variety of music. Other than music heard on radio, they would have had to purchase records, tapes, and CDs. In contrast, because the younger participants have had relatively easy access to a greater variety of music, they could freely sample music of widely different styles and eras, especially that from the preferred decades.

Another generational effect was that the younger participants tended to listen alone less than the older participants. One might have thought, with the availability of personal listening devices, they would be listening alone more. A survey done by Edison Research^6^ found that listeners report friends and family were among the most important sources to keep up-to-date with music, together with AM/FM radio, suggesting they discover music by listening with others. The present finding that younger listeners listen alone less also fits with the idea that music sharing is used to as a way to convey information about ourselves to others (Rentfrow and Gosling, 2003, 2006; Lonsdale and North, 2009).

A somewhat surprising result was that the older participants generally found the music less sad than the younger participants. This may be because the older individuals tend to focus on more positive things in general (Mather and Carstensen, 2005), so that they might have focused on the more upbeat songs in each decade. Alternately, the effect might be specific to music, with older participants having had more experience with the older sadder music and thus responded less to the sad content in the songs while, conversely, the younger participants were more experienced with less happy music and were thus responding to the less happy content. Music is multivalent, in as much as it can express multiple emotions simultaneously (Krumhansl, 1997; Vines et al., 2005), so that the same piece of music might be, for example, happy, sad and nostalgic at the same time.

The final generational effect came from looking at the oldest birth cohort, those born in the 1940s, to see the cumulative effect of listening to music over approximately 70 years. This birth cohort had the most eclectic taste of all the cohorts, that is, they liked music from all periods of their lives except from the last two decades, as will be discussed below. The finding argues against the stereotype of that generation (mostly “baby boomers”) has musical tastes confined to music of the 1960s. Although that music played a strong role in defining their identities, their musical tastes are considerably broader than just the music of their youth.

Schubert’s (2016) younger participants reported their tastes broadening over time. The result for this older generation suggests that this process might continue well into the lifetime. This kind of “open-earedness” (Hargreaves, 1982) may be facilitated by the variety of listening niches the oldest participants have occupied. Listening with parents, siblings and other family members, friends and peers, spouse, or partner, and finally with children have given them broad exposure to, and developed their liking for, music of many decades. Cohen (2000) has suggested reduced plasticity with age makes it difficult to acquire the grammar of new styles of popular music, and this might be reflected in the steep drop off in preferences for the most recent music. It may also be that people in their 60s and 70s no longer typically occupy multigenerational listening niches.

The music industry is currently undergoing rapid changes in how music is produced, delivered, and shared between individuals. What will come of these changes is a question of great interest. If the present findings offer any guidance, various forces are likely to play a stabilizing role in future developments. One is that people move through a generally regular sequence of listening niches that are populated by different individuals and media over time. They adapt to new technologies in a gradual way. Musical tastes tend to broaden with age, and listening to music is a social activity with people sharing music recommendations with one another, increasingly across generations. All these forces, at least as they have operated over the last century, have produced systematic patterns of change over time despite the marked evolution of musical styles and technologies. Rather than creating ruptures in music listening patterns, periods of particularly rapid evolution have in fact resulted in enhanced preferences for, and emotional responses to, music from those decades.

## Author Contributions

CK designed the study, analyzed the results, prepared the figures, and wrote the manuscript.

## Conflict of Interest Statement

The author declares that the research was conducted in the absence of any commercial or financial relationships that could be construed as a potential conflict of interest.

## Footnotes

1 https://www.emarketer.com/Article/Pandora-Maintains-Strong-Audience-Lead-Over-Spotify/1012476

2 http://en.wikipedia.org/wiki/List_of_Billboard_YearEnd_number_one_singles_and_albums#cite_note-221

3 https://www.archive.org

4 http://music.psych.cornell.edu

5 http://www.npr.org/sections/health-shots/2013/09/05/219278386/turns-out-your-kids-really-did-love-that-music-you-played

6 http://www.edisonresearch.com/the-infinite-dial-2016/

## Appendix A

### Songs Used in the Survey

**Table:**

**1910–1919**	
Casey Jones	Billy Murray
Alexander’s Ragtime Band	Arthur Collins and Byron Harlan
Moonlight Bay	American Quartet
When Irish Eyes Are Smiling	Chauncey Olcott
The Song That Stole My Heart Away	Henry Burr
It’s A Long Way To Tipperary	John McCormack
M-o-t-h-e-r (A Word That Means The World To Me)	Henry Burr
Over There	American Quartet
Just A Baby’s Prayer At Twilight	Henry Burr
Till We Meet Again	Henry Burr and Albert Campbell
**1920–1929**	
Dardanella	Ben Selvin and His Orchestra
Wang-Wang Blues	Paul Whiteman
April Showers	Al Jolson
Parade Of The Wooden Soldiers	Paul Whiteman Orchestra
It Ain’t Gonna Rain No Mo	Wendell Hall
The Prisoner’s Song	Vernon Dalhart
Valencia (A Song Of Spain)	Paul Whiteman and His Orchestra
My Blue Heaven	Gene Austin
Sonny Boy	Al Jolson
Tiptoe Through The Tulips	Nick Lucas
**1930–1939**	
Stein Song	Rudy Vallee
El Manicero (The Peanut Vendor)	Don Azipiazu and The Havana Casino Orchestra
Night and Day	Leo Reisman
The Last Round Up	George Olson and His Music
June In January	Bing Crosby
Cheek To Cheek	Fred Astaire
Pennies From Heaven	Bing Crosby
Sweet Leilani	Bing Crosby
A-Tisket A-Tasket	Ella Fitzgerald, Chick Webb
Deep Purple	Larry Clinton
**1940–1949**	
In The Mood	Glenn Miller
Amapola (Pretty Little Poppy)	Jimmy Dorsey and His Orchestra
White Christmas	Bing Crosby
I’ve Heard That Song Before	Harry James and His Orchestra
Swinging On A Star	Bing Crosby
Rum and Coca Cola	The Andrews Sisters
The Gypsy	The Ink Spots
Near You	Francis Craig
Buttons And Bows	Dinah Shore
Riders In The Sky (A Cowboy Legend)	Vaughn Monoroe and His Orchestra
**1950–1959**	
The Tennessee Waltz	Patti Page
Cry	Johnnie Ray and The Four Lads
You Belong To Me	Jo Stafford
Vaya Con Dios (May God Be With You)	Les Paul, Mary Ford
Little Things Mean A Lot	Kitty Kallen
Cherry Pink And Apple Blossom White	Perez Prado
Heartbreak Hotel	Elvis Presley
All Shook Up	Elvis Presley
Volare (Nel Blue Dipinto Di Blu)	Demenico Modugno
The Battle of New Orleans	Johnny Horton
**1960–1969**	
Theme From “A Summer Place”	Percy Faith
Tossin’ And Turnin’	Bobby Lewis
Stranger On The Shore	Mr. Acker Bilk
Sugar Shack	Jimmy Gilmer and The Fireballs
I Want To Hold Your Hand	The Beatles
Wooly Bully	Sam The Sham and The Pharoahs
The Ballad Of The Green Berets	Sgt. Barry Sadler
To Sir With Love	Lulu
Hey Jude	The Beatles
Sugar, Sugar	Archies
**1970–1979**	
Bridge Over Troubled Water	Simon and Garfunkel
Joy To The World	Three Dog Night
The First Time Ever I Saw Your Face	Roberta Flack
Tie A Yellow Ribbon ’Round The Ole Oak Tree	Tony Orlando
The Way We Were	Barbara Streisand
Love Will Keep Us Together	Captain and Tennille
Silly Love Songs	Wings
Tonight’s The Night (Gonna Be Alright)	Rod Stewart
Shadow Dancing	Andy Gibb
My Sharona	Knack
**1980–1989**	
Call Me	Blondie
Bette Davis Eyes	Kim Carnes
Physical	Olivia Newton-John
Every Breath You Take	The Police
When Doves Cry	Prince
Careless Whisper	Wham!
That’s What Friends Are For	Dionne and Friends
Walk Like An Egyptian	Bangles
Faith	George Michael
Look Away	Chicago
**1990–1999**	
Hold On	Wilson Phillips
(Everything I Do) I Do It For You	Bryan Adams
End Of The Road	Boyz II Men
I Will Always Love You	Whitney Houston
The Sign	Ace of Base
Gangsta’s Paradise	Coolio
Candle In The Wind	Elton John
Too Close	Next
Believe	Cher
**2000–2009**	
Breathe	Faith Hill
Hanging By A Moment	Lifehouse
How You Remind Me	Nickelback
In Da Club	50 Cent
Yeah!	Usher featuring Lil’ Jon and Ludacris
We Belong Together	Mariah Carey
Bad Day	Daniel Powter
Irreplaceable	Beyonce
Low	Flo Rida featuring T-Pain
Boom Boom Pow	The Black Eyed Peas

## Appendix B

### Questions on Survey

For each decade (1910s, 1920s, 1930s, 1940s, 1950s, 1960s, 1970s, 1980s, 1990s, and 2000s):

Percent recognized (0–100)
How much do you like these songs? (0–10)
How much do these songs make you feel sad? (0–10)
How much do these songs make you feel happy? (0–10)
How much do these songs make you feel nostalgic? (0–10)
How much do these songs make you feel energized? (0–10)
How much do these songs make you feel romantic? (0–10)
If given the opportunity, would you choose to hear more songs like this?
Are any of these songs associated with personal memories? (Y/N)
  if so:
  How specific are the memories (for example, who you were with, where, when)? (0–10)
  During what period(s) in your life?-Childhood (up to 13 years old) (Y/N)
  During what period(s) in your life?-Teens (13–19 years old) (Y/N)
  During what period(s) in your life?-20s (including college) (Y/N)
  During what period(s) in your life?-30s (Y/N)
  During what period(s) in your life?-40s (Y/N)
  During what period(s) in your life?-50s-65 (Y/N)
  During what period(s) in your life?-Over 65 (Y/N)
  What context(s)?-Listening alone (Y/N)
  What context(s)?-Listening with parents (Y/N)
  What context(s)?-Listening with spouse/partner (Y/N)
  What context(s)?-Listening with children (Y/N)
  What context(s)?-Listening with siblings or other family members (Y/N)
  What context(s)?-Listening with friends or peers (Y/N)

### Demographics

What is your gender? (M/F)
What year were you born?
What year was your mother born?
What year was your father born?
Do you have children?
  if so:
  What year was your first child born?
  What year was your second child born?
  What year was your third child born?
  What year was your forth child born?
What is your nationality?
Are you living in the USA now? (Y/N)
If so, how many years have you lived in the USA?

For each of three periods of life (growing up at home, about 18 - 25, within the last year or so):
How much did you listen to?
  Pop and Rock (hours per week)
  Rhythm and Blues (hours per week)
  Country and Folk (hours per week)
  Classical (hours per week)
  Jazz (hours per week)
  Ethnic and World (hours per week)
  Other (hours per week)
Where did you hear popular music?
  Radio (Y/N)
  Record (Y/N)
  Tape cassette (Y/N)
  Dances and parties (Y/N)
  Concerts (Y/N)
  Heard performed by family and friends (Y/N)
  Played myself (Y/N)
  CDs (Y/N)
  Subscription services (e.g., Spotify, Rhapsody, etc.) (Y/N)
  YouTube (Y/N)
  Internet radio (e.g., Pandora) (Y/N)
  Digital download (e.g., mp3) (Y/N)
  Other (Y/N)
